# Biosynthesis of rhamnolipid by a *Marinobacter* species expands the paradigm of biosurfactant synthesis to a new genus of the marine microflora

**DOI:** 10.1186/s12934-019-1216-8

**Published:** 2019-10-10

**Authors:** Lakshmi Tripathi, Matthew S. Twigg, Aikaterini Zompra, Karina Salek, Victor U. Irorere, Tony Gutierrez, Georgios A. Spyroulias, Roger Marchant, Ibrahim M. Banat

**Affiliations:** 1 School of Biomedical Sciences, Ulster University, Coleraine, BT521SA Northern Ireland UK; 2 Department of Pharmacy, University of Patras, 26504 Patras, Greece; 3 Institute of Mechanical, Process & Energy Engineering, School of Engineering & Physical Sciences, Heriot-Watt University, Edinburgh, EH14 4AS UK

**Keywords:** Biosurfactant, Glycolipid, HPLC–MS, Marine bacteria, *Marinobacter*, Rhamnolipid

## Abstract

**Background:**

In comparison to synthetically derived surfactants, biosurfactants produced from microbial culture are generally regarded by industry as being more sustainable and possess lower toxicity. One major class of biosurfactants are rhamnolipids primarily produced by *Pseudomonas aeruginosa*. Due to its pathogenicity rhamnolipid synthesis by this species is viewed as being commercially nonviable, as such there is a significant focus to identify alternative producers of rhamnolipids.

**Results:**

To achieve this, we phenotypically screened marine bacteria for biosurfactant production resulting in the identification of rhamnolipid biosynthesis in a species belonging to the *Marinobacter* genus. Preliminary screening showed the strain to reduce surface tension of cell-free supernatant to 31.0 mN m^−1^. A full-factorial design was carried out to assess the effects of pH and sea salt concentration for optimising biosurfactant production. When cultured in optimised media *Marinobacter* sp. MCTG107b produced 740 ± 28.3 mg L^−1^ of biosurfactant after 96 h of growth. Characterisation of this biosurfactant using both HPLC–MS and tandem MS showed it to be a mixture of different rhamnolipids, with di-rhamnolipid, Rha-Rha-C_10_-C_10_ being the most predominant congener. The strain exhibited no pathogenicity when tested using the *Galleria mellonella* infection model.

**Conclusions:**

This study expands the paradigm of rhamnolipid biosynthesis to a new genus of bacterium from the marine environment. Rhamnolipids produced from *Marinobacter* have prospects for industrial application due to their potential to be synthesised from cheap, renewable feed stocks and significantly reduced pathogenicity compared to *P. aeruginosa* strains.

## Background

Surfactant compounds possess both hydrophobic and hydrophilic moieties: they can modulate surface and interfacial tensions and are therefore widely utilised in a variety of different industries. Though many of these surfactant compounds are derived synthetically from petrochemical sources, numerous microorganisms have been shown to synthesise surfactant compounds. Surfactant compounds produced from a biological source are termed biosurfactants and are generally viewed as being more sustainable and less toxic than their synthetically derived alternatives [1]. Marine microorganisms have been shown to be able to produce biosurfactants under extreme environments, caused by changes in salinity, increased UV exposure, limited nutrients, fluctuations in temperatures and pH [2–4]. Many marine bacterial species, commonly from oil-contaminated waters, have been reported to produce biosurfactants, and include members belonging to the genera *Alcanivorax*, *Alteromonas*, *Pseudoalteromonas* and *Halomonas* [5–7]. The biosurfactants they produce have the ability to solubilise hydrocarbons from the surrounding environment, which enhances the growth of indigenous bacteria capable of degrading aliphatic and polycyclic aromatic hydrocarbons (PAHs) [8]. These species therefore have tremendous industrial potential especially for application in microbial enhanced oil recovery (MEOR) and bioremediation purposes [7, 9, 10]. Furthermore, biosurfactant produced by psychrophilic marine bacteria are potentially exploitable in industrial processes for the preparation of biological detergents that are active at lower temperatures [11].

Particularly in the oligotrophic conditions of open ocean environments, marine bacteria have evolved to compete for the limited resources available to them. With respect to biosurfactants, they may be produced as secondary metabolites as, for example, to access hydrophobic growth substrates or to directly attack rival bacterial species competing for limited growth and energy sources [12]. In the latter case, biosurfactant compounds could be considered applicable for combatting pathogenic antibiotic-resistant microorganisms [13, 14]. Biosurfactants have also been shown to play an important role in biofilm development, the maintenance of biofilm structure and in substrate adhesion [15]. Abrogating the bacterium’s ability to produce a biosurfactant could therefore disrupt biofilm growth with a multitude of potential applications including reducing infection risk to patients receiving implantable medical devices such as catheters [16, 17].

Biosurfactant compounds possess a wide array of molecular structures and are often classified based on their structure. One of the best studied groups of biosurfactants are the glycolipids, specifically rhamnolipids [10]. Rhamnolipids are composed of one or two rhamnose units linked in a 1,2-glycosidic linkage to two *β*-hydroxy fatty acids (*β*-OH-FA or 3-OH-FA) ranging between 8 and 18 carbons in length. The most studied rhamnolipids are those produced by the Gram negative, opportunistic pathogen *Pseudomonas aeruginosa* [18]. Rhamnolipids have a broad range of potential applications in various industries, including for MEOR in the petroleum industry, as emulsifiers in the food and cosmetic industries, and as anti-microbial/therapeutically-active agents in the pharmaceutical industry [19]. Despite their versatile potential industrial applications, the exploitation of rhamnolipids has been limited due to the pathogenic nature of *P. aeruginosa*. To overcome this there has been an increased interest in the discovery of non-pathogenic rhamnolipid producers. Recent reports have shown rhamnolipid production by non-pathogenic species of *Pseudomonas*. For example, a non-pathogenic rhamnolipid-producing marine *Pseudomonas* sp. MCTG214(3b1) was shown to produce both mono and di-rhamnolipids [20]. An arctic marine bacterium identified as *Pseudomonas fluorescence* species was reported to synthesise five mono-rhamnolipid congeners [21]. Outside of the *Pseudomonas* genus, rhamnolipids have been shown to be synthesised by a number of non-pathogenic species of the genus *Burkholderia* [22, 23]. Discovery and isolation of novel non-pathogenic rhamnolipid producers is an attractive route to compete with the synthetic surfactants and meet future global biosurfactant requirements. Global estimates of microbial cell abundances in seawater range from 10^4^ to 10^7^ cells/mL, with an estimated average taxonomic diversity of 1000 species/mL [24]. Collectively, this offers a significant opportunity to discover novel biosurfactant producers, including that produce rhamnolipids, and that are non-pathogenic [25]. Importantly for commercial exploitation, it is essential that the economics underlying the production of the biosurfactants is viable and able to compete with chemically-derived surfactants in the global market. This needs to be achieved by reducing manufacturing costs and enhancing fermentation yields. There are numerous factors for optimising the process of biosurfactant production, including optimising the composition of the culture medium, pH, dissolved oxygen levels during growth, culture agitation and incubation temperature. Statistical design of experiments (DoE) methods, such as full factorial design (FFD), have been shown to be an efficient and useful method to optimise biosurfactant production using a reduced number of experiments [26–28].

In this study, we investigated biosurfactant production in five bacterial strains isolated from coastal and offshore sites in the USA, Scotland and Norway, and all phylogenetically identified to belong to the genus *Marinobacter*. One of these strains, *Marinobacter* sp. MCTG107b, possessed phenotypic traits indicative of biosurfactant synthesis. The bioprocess factors for biosurfactant production in this strain were optimised for maximum production yield in shake-flask culture using FFD. Using high performance liquid chromatography–mass spectrometry (HPLC–MS) and tandem-MS, the chemical structure of the biosurfactants produced by this strain were analysed and confirmed to be rhamnolipids. This study reports the first description of rhamnolipid production by a *Marinobacter* and, importantly also, extends the paradigm of rhamnolipid production to a new bacterial genus which is recognised as ubiquitous in the marine environment and commonly associated with oil spills.

## Materials and methods

### Strains and culture conditions

The marine bacteria used in this study were isolated from surface seawater samples collected from offshore location in the USA, UK and Norway (Table 1). The method of isolation has been previously described by Twigg et al. [20]. Following isolation, these strains were routinely cultured at 30 °C in ZM/1 medium which consists of 30 g L^−1^ sea salts (*Sigma*-*Aldrich*), 5 g L^−1^ Bacto Peptone (*BD Biosciences*), 1 g L^−1^ yeast extract (*Sigma*-*Aldrich*) and supplemented with trace elements and vitamins after autoclaving [29]. *P. aeruginosa* strain PAO1 was purchased from the *ATCC* (ATCC 15692) and was cultured at 37 °C in Nutrient Broth (*Oxoid*). Solid media plates used in this study were composed of appropriate culture media supplemented with 1.5% (w/v) agar (*Sigma*-*Aldrich*).

**Table 1:** Phylogenetic identification and biosurfactant phenotypic screening results for each bacterial strain

Strain	Origin	BLASTn identification (against NCBI database)	GeneBank accession number	Sequence similarity (%)	Phenotypic screening	
					ST (mN m^−1^)^a^	EI_24 h_ (%)^b^
MCTG106	Coastal surface water, Oregon, Washington State, USA	*Marinobacter* sp. NP1383C-30R	MK894600	100	54.63 ± 3.5	40 ± 1.5
MCTG4b	Laboratory culture of *Thalassiosira weissflogii* strain CCMP 1052 isolated from Oslo Fjord, Norway	*Marinobacter* sp. Set72	MK894835	99	38.5 ± 0.6	42 ± 2.0
MCTG167	Phytoplankton net tow, Oban, UK	*Marinobacter* sp. T23	MK894854	100	61.55 ± 0.1	N/A
MCTG161(2c3)	Phytoplankton net tow, Oban, UK	*Marinobacter adhaerens* HP15	MK894872	99	60.0 ± 0.5	45 ± 2.0
MCTG107b	Coastal surface water, Oregon, Washington State, USA	*Marinobacter* sp. R-28768	MK578516	100	31.0 ± 0.5	40 ± 1.8

Bacteria were identified by 16S rDNA gene sequencing. Surface tension values (mN m^−1^) and EI_24_ (%) were obtained from cell-free supernatant samples of cultures incubated for 96 h

^a^ST and ^b^EI 24 h of sterile ZM/1 medium was 58 mNm^−1^ and 0%, respectively

### Phylogenetic identification

Genomic DNA (gDNA) was extracted from approx. 1 × 10^8^ bacterial cells via a DNeasy Blood and Tissue Kit (*Qiagen*) used as per the manufactures instructions for Gram-negative bacteria. Extracted gDNA was quantified and assessed for purity by measuring absorbance at 260 nm and 280 nm using a Nanodrop 2000 spectrophotometer (*Thermo Fischer*). The 16S rRNA gene was then amplified using the Polymerase Chain Reaction (PCR) with the universal primers 9bfm and 1512uR. PCR reactions contained 50 ng of gDNA, 1.5 mM MgCl_2_, 1× PCR buffer (*Thermo Fischer*), 0.2 mM dNTP mix (*Thermo Fischer*), 0.5 mM of each primer (*Thermo Fischer*), and 2 U of *Taq* DNA polymerase (*Thermo Fischer*). The PCR reaction was as follows: one cycle initial denaturation at 94 °C for 3 min; 30 cycles of denaturation at 94 °C for 45 s; annealing step at 52 °C for 30 s; extension step at 72 °C for 90 s; and one cycle final extension at 72 °C for 5 min. Following amplification, PCR products were separated on a 1% (w/v) agarose gel made with TBE buffer (*Thermo Fischer*), and amplicons of approx. 1.5 kb were subsequently purified from the gel using a Wizard SV Gel and PCR Clean Up System (*Promega*). Amplified 16S rDNA was quantified and assessed for purity as above. The purified 16S rDNA was sequenced using the Sanger method by *Eurofins Genomics* (Cologne, Germany) with primers 9bfm, 536F, 907R and 1512uR [30, 31]. The resultant DNA sequences were compared to the NCBI nucleotide database using BLASTn.

### Phenotypic screening for biosurfactant production

The five marine bacterial strains were screened for their ability to reduce surface tension and to emulsify oil in water. Bacterial cultures were centrifuged at 13,000×*g* for 15 min and the supernatant fractions (in triplicate) used to perform surface tension measurements at room temperature (21 °C) according to the Du Noüy ring method using a K10ST A KRÜSS KIOT Tensiometer (*Krϋss*) [32]. The surface tension of sterile ZM/1 media supplemented with 1% (v/v) rapeseed oil (*Sigma*-*Aldrich*) was also measured as a comparative control. To evaluate for emulsification, the emulsification index (EI) of the supernatant fractions was measured by adding 2 mL of the supernatant to an equal volume of kerosene and vortexing at high speed for 2 min. The stability of resultant emulsions was observed after 24 h settlement. The EI_24_ (i.e. EI after 24 h) was calculated as a percentage of the height of the emulsified layer to the total height of the liquid prior to emulsification by vortexing [33]. As a control for comparison, sterile ZM/1 medium was used.

### Optimization of growth conditions

The growth of MCTG107b in different physical and media conditions was investigated. For this, shake flask experiments were carried out in 1 L Erlenmeyer flasks containing 90 mL of ZM/1 medium and inoculated (10% v/v) with a seed culture grown under the standard conditions described earlier. For the carbon source, glucose 1% (w/v) final concentration was used in all experiments. All flasks were incubated in a rotary orbital incubator set at 200 rpm. However, physical and media conditions tested included various concentrations of sea salts (5, 10, 20, 30 and 40 g L^−1^), temperature (25, 28, 30 and 37 °C) and pH (4.0, 5.5, 7, 8.5). Samples of the culture medium in these various experiments were taken at various time points for optical density (OD) measurements at 600 nm to monitor the growth of the cells.

### Optimisation of growth media for biosurfactant production

The effect of sea salt concentration and pH for enhancement of biosurfactant production by MCTG107b was carried out by FFD, using surface tension, (measured as described previously), and biosurfactant yield, (measured gravimetrically), as response variables. These experiments were carried in shake flask culture using ZM/1 medium supplemented with 1% (v/v) rapeseed oil with various concentrations of sea salts (5 to 40 g L^−1^) and pH (5.5 to 8.5) according to the experimental designs (Additional file 1: Table S1). In total, 14 experiments were performed (2^2^ FFD with 8 assays and 6 replicates at the centre point).

### Biosurfactant extraction and purification

Biosurfactant compounds were extracted and purified from 3-L cultures of strain MCTG107b when grown in optimised ZM/1 medium supplemented with 1% (v/v) rapeseed oil using a 5.0 L Biostat B bioreactor (*Sartorius Stedim*) equipped with a mechanical foam separator. The reactor vessel was inoculated (10% v/v) with a MCTG107b seed culture grown to exponential phase in ZM/1 supplemented with 1% (w/v) glucose at 30 °C and 200 rpm. Internal temperature of the culture was maintained at 30 °C throughout the growth cycle. Stirrer speed and aeration varied between 300 and 600 rpm in order to maintain DO_2_ levels at 50%. Cultures were incubated for 96 h; during growth, dissolved oxygen and pH were continually monitored and samples were taken at 24 h intervals to monitor growth and BS.

At the termination of the culture (96 h), the biosurfactants were extracted using liquid phase extraction. For this, the culture volume was first centrifuged (13,000×*g*; 15 min) and then the supernatant collected and acidified to pH 2.0 with 1 M HCl (*Sigma*-*Aldrich*) prior to extraction three times with an equal volume of ethyl acetate (*Sigma*-*Aldrich*). The organic phase was separated and dried using MgSO_4_ (*Sigma*-*Aldrich*), filtered and rotary evaporated under vacuum at 40 °C to obtain a crude extract [34]. Crude extracts were then purified by Solid Phase Extraction (SPE) using Strata SI-1 Silica (55 μm, 70 Å) Giga tubes (*Phenomenex*). Purified BS extracts were gravimetrically assessed and stored at 4 °C for further analysis [35].

### Chemical analysis of biosurfactant compounds

Biosurfactant compounds extracted from strain MCTG107b were initially analysed using the orcinol method [34]. To each 100 μL sample, 900 μL of a solution containing 0.19% (w/v) orcinol (*Sigma*-*Aldrich*) in 53% H_2_SO_4_ (*Sigma*-*Aldrich*) was added. Samples were then heated to 80 °C for 30 min, after which the samples were cooled to room temperature. The absorbance of the samples was measured at 421 nm. The concentration of glycolipid present in the samples were calculated to those generated using a standard rhamnose at concentrations of 0–100 μg mL^−1^ [36].

Individual biosurfactant congeners were identified in the SPE purified extract by a UHPLC system with RS Diode Array detector (*ThermoFisher Scientific*) in conjunction with the amaZon SL dual funnel Ion Trap spectrometer LCMS system (Bruker). An analytical column of Acclaim RSLC, 120 C18, 2.2 μm 120 Å (2.1 × 100 mm) (*ThermoFisher Scientific*) was used for analysis. The gradient elution sequence used was as follows: 20% B to 100% in 30 min, 100% B for 10 min, 20% B in 5 min. Solvents A = H_2_O (0.1% TFA), B = AcCN (0.1%TFA). The sample injection volume used was 10 μL. Spectra were acquired in the positive mode from *m/z* 200 to 2000. Tandem-MS was performed using Thermos System LC P4000 (*ThermoFisher Scientific*) coupled to a LCQ classic MATT ion trap mass spectrometer (*ThermoFisher Scientific*) equipped with a 150 × 4.6 mm Kinetex 5 µM F5 100 Å LC column. HPLC-grade water and analytical-grade acetonitrile were used as mobile phase. The sample injection volume was 5 µL and the spectra were acquired in the negative mode from *m/z* 175 to 700. The fragmentation of the molecules was done with helium gas at the normalised 40% collision energy with the activation q value of 0.25.

### *Galleria mellonella* infection model

Virulence assessment of strain MCTG107b was carried out using the *G. mellonella* infection model [38]. A comparative positive control for these experiments was *P. aeruginosa* PAO1 (ATCC 15692). Using the Miles and Misra method [37], viable count (CFU) and OD 600 nm were correlated throughout the growth cycles of both strains. Following this, 10 mL of stationary phase culture was centrifuged (10,000×*g*; 20 min) and the pelleted cells washed in sterile phosphate buffered saline (PBS). The washed cells were re-suspended to OD 600 nm 0.4 in PBS to a concentration of 5 × 10^4^ CFU mL^−1^. *G. mellonella* larvae (*Pets at Home*, Belfast) of approx. 20 mm in length and 200 mg in weight were selected and 20 μL of either bacterial sample (1000 CFU) or PBS (negative control) was injected into the posterior pro-leg of individual larvae (n = 10 per experimental group). Injection was carried out using a 0.30 mm (30G) × 8 mm hypodermic needle (BD). Immediately following injection, the larvae were incubated at 37 °C and observed at set time points during the course of a 48 h period. At each time point, individual larva was recorded as either live or dead. The experiment was performed on three independent occasions which gives a total of n = 30 per experimental group [20, 38].

### Statistical analysis and data availability

Statistical analysis of bacterial growth experiments was carried out in GraphPad Prism V.7 using a one-way ANOVA followed by Tukey’s post hoc testing; the significance of the results was tested at *p* < 0.05 level. The data obtained from FFD experiments were subjected to statistical analysis by TIBCO Statistica software version; the significance of the results was tested at *p* < 0.05 level. All sequence data was submitted to GenBank (NCBI, USA) and the assigned accession numbers of strains are given in Table 1.

## Results

### Strain identification and initial phenotypic screening

Five marine bacterial isolates—MCTG107b, MCTG4b, MCTG106, MCTG167 and MCTG161(2c3)—were investigated for biosurfactant production. BLASTn analysis of partial 16S rRNA gene sequences from these strains showed > 99% similarity to the genus *Marinobacter* (Table 1). Following phenotypic screening, strains which reduced the surface tension of culture medium to below 35 mN m^−1^ and/or produced a stable emulsion after 24 h were considered as potential biosurfactant(s) producers (Table 1). Strain MCTG167 was unable to form a stable emulsion or to significantly reduce the surface tension when compared to un-inoculated medium controls (58 mN m^−1^). Strain MCTG106 and MCTG161(2c3) also showed no significant reduction in surface tension, however both strains were able to form stable emulsions after 24 h. Strain MCTG4b emulsified kerosene with an EI_24_ of 40%, whereas it reduced the ST of supernatant fractions to 38.5 mN m^−1^. Strain MCTG107b showed the highest surface activity, significantly reducing the surface tension of the culture broth to 31 mN m^−1^, and producing stable emulsions with kerosene (EI_24_ of 40%). Therefore, *Marinobacter* sp. MCTG107b was selected for further study.

### Bacterial growth of *Marinobacter* sp. MCTG107b

To determine optimal conditions for the growth of *Marinobacter* sp. MCTG107b, and with a view to optimising its production of biosurfactant, growth was monitored in different physical and media conditions that included evaluating different salinities, pH, temperatures and nitrogen sources. *Marinobacter* sp. MCTG107b grew optimally at a range of salinity concentrations, from 5.0 to 40 g L^−1^ sea salts. In medium containing no added sea salts, no growth was observed. Strain MCTG107b grew optimally within a pH range of 5.5 to 8.5, whereas it was significantly inhibited under more acidic conditions (pH 4.0). The strain grew optimally at temperatures ranging from 25 to 37 °C (Additional file 1: Fig. S1).

### Optimization of culture conditions for biosurfactant production

To optimise the media composition for maximal biosurfactant production by strain MCTG107b, the effects of salinity and pH, as well as the interaction between these variables was assessed by applying FFD, 2^2^. Compared to pH, salinity was observed to be the most important factor affecting the reduction of surface tension when this was measured for cell-free culture supernatant fractions. The surface tension was found to vary between 30.5 to 40.2 mN m^−1^ (Additional file 1: Table S1). Experimental results were used to generate two equations that modelled the relation between pH/salinity and the outputs biosurfactant yield and surface tension. According to the response values obtained from the designed experiments, the following regression equations were obtained for both biosurfactant yield (1) and surface tension (2):

1 $$Yield = 22.17262 + 39.88095\;*\;pH\left( 1 \right) + 36.60714\;*\;Salts\left( 2 \right) - 4.64286\;*\;pH\;*\;Salts\left( {1 \;by\; 2} \right)$$

2 $$Surface\;Tension = 50.03036 - 1.70714\;*\;pH\left( 1 \right) - 0.55690\;*\;Salts\left( 2 \right) + 0.05476\;*\;pH\;*\;Salts\left( {1\;by\;2} \right)$$

**Fig. 1:**
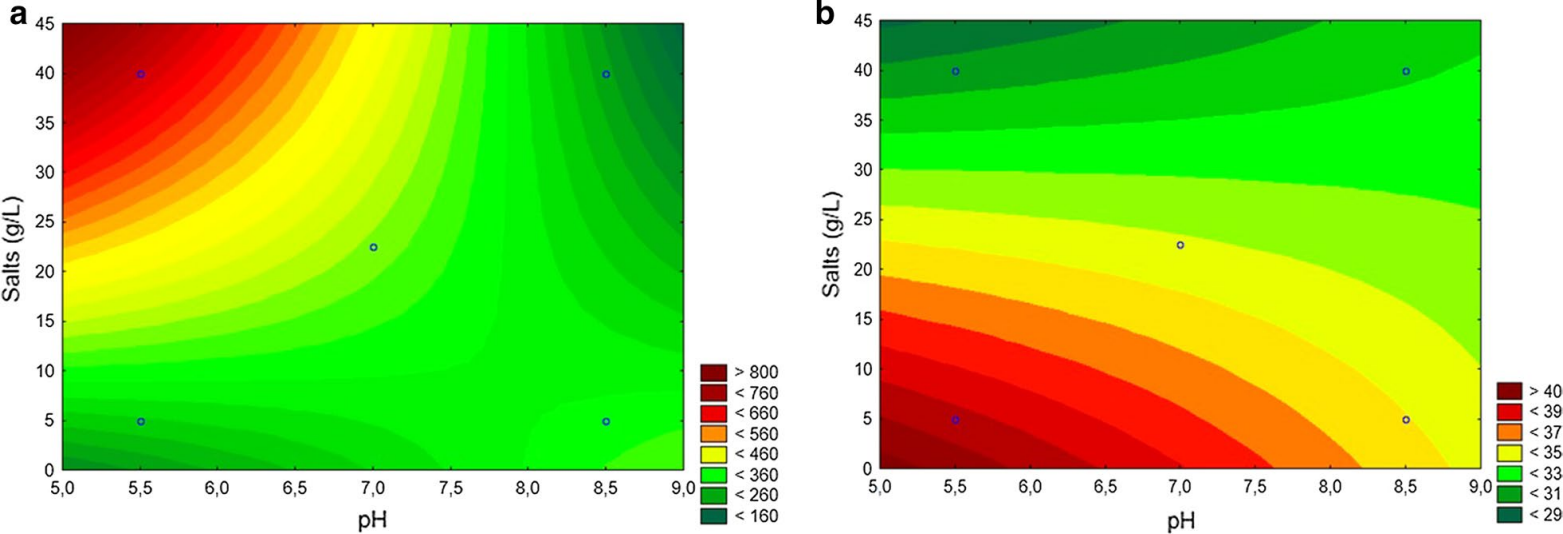
Three-dimensional response surface plot modelling the effect of varying media pH and salt concentration on **a** biosurfactant yield and **b** cell-free supernatant surface tension. The different coloured areas of these plots represent various bands for either predicated yield or predicted surface tension. The values of each band are provided in the key next to each panel

Significance of the present model was validated through analysis of variance (*p* ≤ 0.05) (Additional file 1: Table S2). The observed values for biosurfactant yield were significantly close to those determined by the model (R^2^ = 0.996). The observed and the predicted values for surface tension modelled here also demonstrated that the experimentally observed values were significant to those determined by the model (R^2^ = 0.958). Results from the FFD analysis for the outputs of biosurfactant yield and surface tension were expressed as 3D response surface plots showing the relationship between independent and dependent variables (Fig. 1). Increased salinity significantly and positively influenced biosurfactant yield. Our model demonstrated a significant relationship between biosurfactant yield and the two variables tested (salinity and pH). We modelled an increased biosurfactant yield when pH was low and salinity was increased from the central to the highest level. The increase in salinity positively influenced biosurfactant yield, in a statistically significant way (Fig. 1a). Similarly, the model showed that an increase in pH did not cause major impacts on surface tension, whereas an increase in salinity from the central to highest point led to a decrease in surface tension. The results of reduction in superficial tension agree with the biosurfactant yield. However, the 3D response surface plots of both dependent responses are not a coincident. Since, the increase in salinity concentration negatively influenced, in a statistically significant way, the increase in biosurfactant production, leading to lower surface tension (Fig. 1b). These data demonstrate salinity and pH are critical factors that markedly affected the production yield of biosurfactant. Our model showed maximum production of biosurfactant at a pH 5.0–6.8 and salinity 22.5–40.0 g L^−1^, resulting in a predicted yield of between 460 and 800 mg L^−1^. The optimised medium with a sea salt concentration of 30 g L^−1^ and pH 6.5 was therefore chosen for all subsequent experiments with strain MCTG107b.

### Growth and biosurfactant production by *Marinobacter* sp. MCTG107b in a bioreactor

**Fig. 2:**
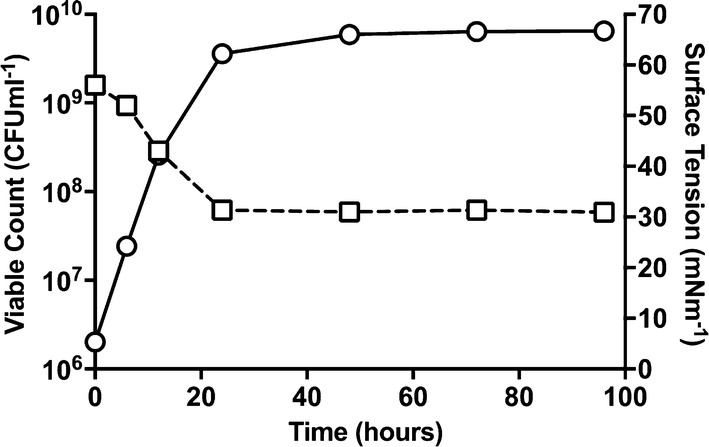
Biomass and surface tension reduction kinetics of *Marinobacter* sp. MCTG107b during growth under optimised conditions using 1% (v/v) rapeseed oil as a carbon source in a 5 L bioreactor. Surface tension (open square) was seen to reduce to a stable value within the first 24 h of growth and corresponded with the strain reaching the stationary growth phase, as measured by viable cell counts (open circle)

Based on our results above, an optimised culture medium was used to produce biosurfactant from *Marinobacter* sp. MCTG107b employing a bench-scale 5.0 L bioreactor. Biomass, culture pH and surface tension were monitored continually throughout the growth cycle. The cell concentration at the time of inoculation (t = 0 h) was 2.02 × 10^6^ CFU mL^−1^. An exponential growth phase was maintained for the first 24 h, followed by a stationary phase from 24 to 96 h, and the cell concentration reaching 6.46 × 10^9^ CFU mL^−1^ by the end of the fermentation (Fig. 2). The pH of the culture was observed to fall during the course of the exponential growth phase and then remained moderately constant during the stationary phase. A similar pattern was observed for the surface tension that was measured for cell-free supernatant samples over the course of the growth phase. The strain achieved the lowest surface tension value (31 ± 0.7 mN m^−1^) after 24 h of fermentation and then remained almost constant until the end of the fermentation (Fig. 2). When assessed gravimetrically, the mean biosurfactant yield obtained by liquid phase extraction and SPE purification of the cell-free supernatant volume from replicate 96 h bioreactor cultures of *Marinobacter* sp. MCTG107b was 740 mg L^−1^ (± 28.3 mg L^−1^).

### Chemical characterisation of the biosurfactant produced by *Marinobacter* sp. MCTG107b

**Fig. 3:**
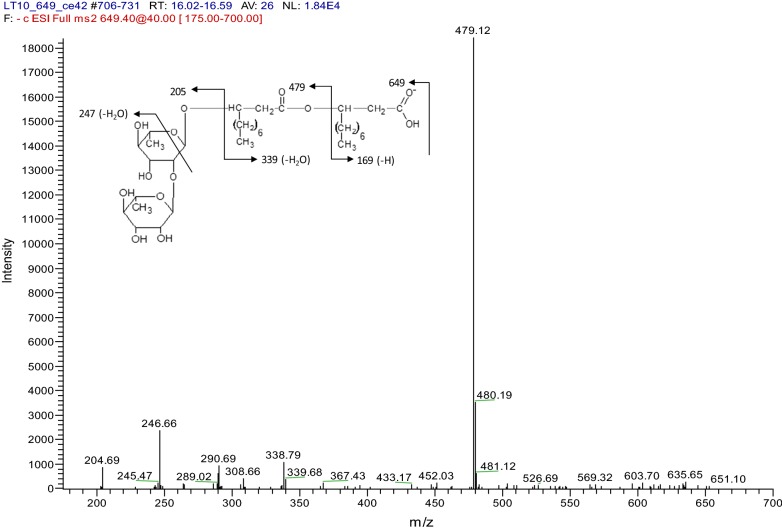
HPLC–MS–MS profile of daughter products resulting from the fragmentation of a molecular ion with an *m/z* of 651.73, observed in a previous HPLC–MS analysis to be the predominant compound in supernatant extracts from *Marinobacter* sp. MCTG107b. The observed products corresponded to the predicted molecular weights of the fragmentation of di-rhamnolipid Rha-Rha-C_10_-C_10_. Fragments below *m/z* 205 were not detected due to sensitivity of the instrument

The traits measured for biosurfactant production (i.e. surface tension reduction and emulsification) were indicative that *Marinobacter* sp. MCTG107b produces a glycolipid biosurfactant. To confirm this, Orcinol assays followed by HPLC–MS analysis were carried out on samples obtained from the bioreactor cultures. Orcinol assays performed with cell-free supernatant samples indicated the presence of glycolipids at 150 µg mL^−1^ of culture. The production and presence of glycolipids was further investigated by mass spectrometric analysis. The identification of glycolipid congeners produced by strain MCTG107b was characterized by HPLC–MS operating in the positive mode. The observed products possessed *m/z* values that corresponded to values for known rhamnolipids, indicating that the biosurfactant synthesised by strain MCTG107b was a mixture of rhamnolipid congeners. We identified a variety of separate rhamnolipid congeners present in purified cell-free supernatant extracts from culture samples of the strain (Table 2). These congeners included both mono- and di-rhamnolipids; however, there was an overwhelming preference toward the synthesis of di-rhamnolipid (95.39% of total rhamnolipid abundance). The congener with the highest relative abundance (52.45%) possessed an *m/z* value of 651.73. This value correlated with *α*-l-rhamnopyranosyl-*α*-l-rhamnopyranosyl-*β*-hydroxydecanoyl-*β*-hydroxydecanoate (Rha-Rha-C_10_-C_10_) with a molecular weight of 650.79 Da. The next most abundantly synthesised congeners were Rha-Rha-C_10_-C_10_CH_3_ (23.07%), Rha-Rha-C_10_ (5.13%), Rha-Rha-C_10_-C_12_ (5.01%), Rha-Rha-C_10_-C_12_CH_3_ (3.26%) and Rha-C_14:2_ (3.18%) (Table 2). As rhamnolipid production has not been previously observed in any member of the *Marinobacter* genus, these data were further investigated for confirmatory evidence of this. For this, tandem-MS was performed on the major molecular ion shown to be synthesised by strain MCTG107b. Tandem-MS analysis of the compound with an *m/z* of 651.73 revealed the detection of ‘daughter’ ions with molecular weights indicative of the fragmentation of Rha-Rha-C_10_-C_10_ (Fig. 3). These data, together with the phenotypic results and initial HPLC–MS and Nuclear Magnetic Resonance (NMR) spectroscopy analysis (data not shown), confirms the synthesis of rhamnolipid by *Marinobacter* sp. MCTG107b.

**Table 2:** Composition of rhamnolipid congeners synthesised by *Marinobacter* sp. MCTG107b

RT min	*m/z* value	Compound	Mw (Da)	Molecular form	Relative %
Mono-rhamnolipid congeners					
14.8	387.22	Rha-C_14:2_	386.48	C_20_H_34_O_7_	3.18
21.5	533.46	Rha-C_10_-C_12_/Rha-C_12_-C_10_	532.71	C_28_H_52_O_9_	0.22
24.2	503.47	Rha-C_10_-C_10:1_	502.64	C_26_H_46_O_9_	0.27
26.9	561.52	Rha-C_12_-C_12_/Rha-C_10_-C_14_	560.76	C_30_H_56_O_9_	0.94
Subtotal					4.61
Di-rhamnolipid congeners					
4.6	453.27	Rha-Rha-C_8_	452.49	C_20_H_36_O_11_	1.95
12.7	480.39	Rha-Rha-C_10_	480.55	C_22_H_40_O_11_	5.13
22.1	537.45	Rha-Rha-C_14_	536.65	C_26_H_48_O_11_	0.21
31.0	649.71	Rha-Rha-C_10_-C_10:1_/Rha-Rha-C_10:1_-C_10_	648.74	C_32_H_56_O_13_	2.85
32.1	651.73	Rha-Rha-C_10_-C_10_	650.79	C_34_H_58_O_13_	52.45
32.8	677.77	Rha-Rha-C_10_-C_12:1_	676.83	C_33_H_60_O_13_	1.06
33.0	665.77	Rha-Rha-C_10_-C_10_-CH_3_	664.82	C_42_H_60_O_13_	23.07
34.5	803.54	Decenoyl-Rha-Rha-C_10_-C_10:1_	801.01	C_35_H_72_O_11_	0.40
35.1	679.78	Rha-Rha-C_10_-C_12_/Rha-Rha-C_12_-C_10_	678.84	C_35_H_64_O_13_	5.01
37.2	693.90	Rha-Rha-C_10_-C_12_-CH_3_/Rha-Rha-C_12_-C_10_-CH_3_	692.80	C_35_H_64_O_13_	3.26
Subtotal					95.39

Rhamnolipid congeners were identified via HPLC–MS in SPE purified extracts from cell-free culture supernatant samples obtained after 96 h growth in a bioreactor

### Assessment of virulence using the *Galleria mellonella* infection model

**Fig. 4:**
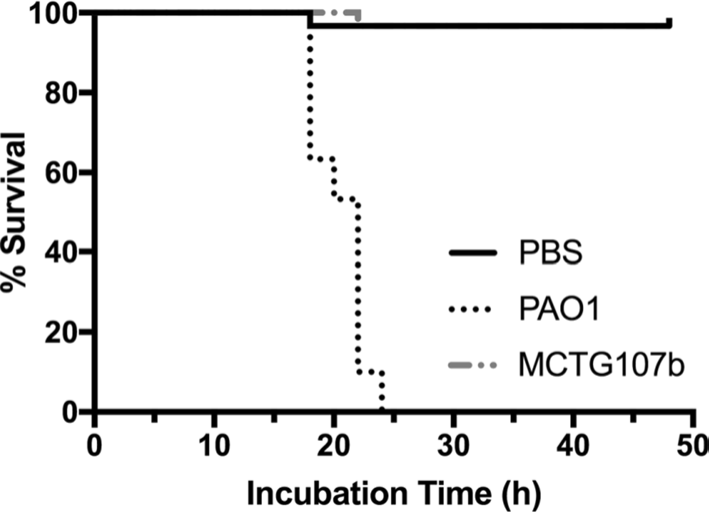
Kaplan–Meier plot showing percentage survival of *Galleria mellonella* larvae after inoculation with either *Marinobacter* sp. MCTG107b or *P. aeruginosa* PAO1. Within a 48 h incubation there was no significant mortality observed after infection with cells of strain MCTG107b as opposed to infection with strain PAO1 where 100% mortality was observed following 24 h incubation. Additionally, no significant mortality was observed in larvae inoculated with the carrier control buffer (PBS). n = 30 (pooled from 3 × duplicate experiments)

The potential virulence of *Marinobacter* sp. MCTG107b was assessed and compared to that of the rhamnolipid producing opportunistic pathogen *P. aeruginosa* using the *G. mellonella* infection model. *P. aeruginosa* PAO1 killed 100% of the infected larvae 24 h post inoculation with as little as 1000 CFU. When larvae were inoculated with an equal CFU count of *Marinobacter* sp. MCTG107b or with sterile PBS, the larvae showed 97% survival 48 h post inoculation, with only one larva dying in each experimental group at 22 and 20 h post inoculation respectively (Fig. 4). Similar survival rates were also observed when larvae were inoculated with significantly higher doses of *Marinobacter* sp. MCTG107b (up to 10,000 CFU in 20 µL) (data not shown).

## Discussion

Marine bacteria are reported to secrete surface-active molecules that can interact with hydrocarbons to increase the emulsification of the hydrocarbon molecules in seawater to enable these, and also non-biosurfactant producing bacteria to access these molecules for uptake and use as a source of carbon and energy [39, 40]. *Marinobacter*, a genus of *Gammaproteobacteria*, has previously been shown capable of utilising hydrocarbons as growth substrates by producing biosurfactants or bioemulsifier [41, 42]. In the present study, five marine bacterial strains, which were originally isolated for their ability to grow on and degrade PAHs, were identified to belong to the genus *Marinobacter* based on 16S rDNA gene sequencing. These isolates were screened to evaluate their potential as biosurfactant producers when cultivated in marine media using rapeseed oil as a carbon source. Many previous studies have reported that the inclusion of peptone in marine media is essential for biosurfactant synthesis [43, 44]. Here we show that in response to adding rapeseed oil to peptone containing ZM/1 media, these five *Marinobacter* strains displayed varying phenotypic responses which were indicative of biosurfactant synthesis. Phenotypic comparison of all these strains showed that *Marinobacter* sp. MCTG107b, isolated from sea surface water samples off the coast of Oregon, USA, showed maximum reduction in the surface tension of cell-free supernatant fractions. As the ability to reduce the surface tension of cell-free supernatant is a key phenotypic marker of low-molecular weight biosurfactant synthesis, this strain was selected for further investigation [45].

Considering that subtle changes in salinity and pH can exert an important effect on microbial communities in the marine environment [46, 47], these parameters were tested for their influence on the growth of strain MCTG107b and its production of biosurfactant. Indeed, several studies have shown the effect of these two parameters on bacterial biosurfactant synthesis. For example, *Bacillus subtilis* N3-1P, isolated from brewery waste, reduced the surface tension of the culture medium to the greatest extent at a pH of 6.41 when compared to a range of other media pHs [48]. A thermophilic and halo-tolerant strain of *P. aeruginosa*, isolated from oil-contaminated soil, produced biosurfactant when cultured in media containing a salinity range of 0–6% (w/v) [49]. In contrast *Bacillus licheniformis* BAS50, isolated from a deep oil well, produced biosurfactant when using a salinity of up to 13% NaCl, which is a salinity equivalent to that present in many petroleum reservoirs [50]. Based on these previous studies, we carried out FFD modelling to identify optimal concentrations of salinity and pH for the maximal production of biosurfactant by strain MCTG107b, and which revealed that a pH range of 5.0–6.8 and salinity concentration of 22.5–40 g L^−1^ were optimal. Interestingly, while salinity was the dominant factor affecting the reduction of surface tension, the strain preferred an acidic pH for increased biosurfactant production. This was at the expense of bacterial growth, indicating that when the culture medium was alkaline, cells were directed to the production of cellular biomass over biosurfactant synthesis. Whilst not within the focus of this study, future work could be directed to explore this pH-mediated biosurfactant response and whether it is transposable to the global ocean in order to predict microbial biosurfactant production under future climate change conditions, such as ocean acidification. The profile of a marked fall in surface tension during the exponential growth phase coupled with a sustained low surface tension during stationary phase indicated the biosurfactant produced by strain MCTG107b is likely a secondary metabolite, as has been similarly reported for *P. aeruginosa* [51], *B. thailandensis* [23] and marine *Pseudomonas* sp. MCTG214(3b1) [20].

Orcinol assay yielded 150 μg mL^−1^ of rhamnolipid in the cell free supernatant. Orcinol assay is a colorimetric method for the rapid indication of rhamnolipid in the fermentation broth. However, the limitation of orcinol method is that, it is not specific for rhamnose and impurities present in the sample might interfere with the actual yield. We followed the criteria suggested by Irorere et al. [35] to quantify biosurfactant yield gravimetrically. The final yield of rhamnolipid was 740 ± 28.3 mg L^−1^ from the SPE purified sample. SPE purification of crude biosurfactant removed excess lipids from the sample which gave an absolute quantification of rhamnolipid yield. Similarly, Perfumo et al. [51] reported purified rhamnolipid yields in the range of 0.8–1.7 g L^−1^ from *P. aeruginosa* strains. While, the orcinol assay provided an overestimation of rhamnolipids at 7.7–9.5 g L^−1^.

The chemical characterisation of the purified biosurfactant synthesised by strain MCTG107b was achieved using HPLC–MS—a methodology demonstrated to be the most effective for identifying biosurfactant compounds [35]. We identified 14 separate rhamnolipid congeners with Rha-Rha-C_10_-C_10_ (*m/z* 651) being the most abundant (52.45%). Furthermore, elucidation of this major molecular ion synthesised by *Marinobacter* sp. MCTG107b was performed by tandem-MS. When fragmented by MS–MS, the parent ion showed characteristic fragments at *m/z* 479 and *m/z* 339, which were in agreement with the previous tandem-MS analysis of Rha-Rha-C_10_-C_10_ performed by Zhao et al. [52]. The pattern of rhamnolipid congeners synthesised by this strain was highly similar to the rhamnolipid congeners that have been shown to be produced by both *P. aeruginosa* and marine *Pseudomonas* sp. MCTG214(3b1), but contrasts with *B. thailandensis* which produces an abundance of congeners with di-rhamnolipid containing C_14_ [18, 20, 23]. The composition of rhamnolipid congeners greatly affects its properties. Mono-rhamnolipids have been reported to more effectively solubilise PAHs compared to di-rhamnolipids. However, di-rhamnolipids have better rate of biodegradation than mono-rhamnolipids due to the slow release of PAH from the mono-rhamnolipid micelles [53]. This was seen with methyl esters of di-rhamnolipid which were reported to be effective in promoting alkane degradation [54]. Interestingly, non-ionic rhamnolipids or methyl esters of di-rhamnolipid C_10_-C_10_ and novel methyl ester of di-rhamnolipid C_10_-C_12_ were also identified in this study. In this study a mono-rhamnolipid with single 3-hydroxy fatty acid chain Rha-C_14:2_ (3.18%) was also detected which was previously reported in rhamnolipid produced by *P. aeruginosa* mutant MIG-N146 [55]. Under our experimental conditions, MCTG107b was able to produce diverse rhamnolipid congeners with aliphatic chains varying from C_8_ to C_14_ and few congeners with unsaturated bonds.

The biosynthesis of rhamnolipids in *P. aeruginosa* occurs in three enzymatic steps. In the first step, rhamnosyltransferase chain A RhlA (encoded by the *rhlA* gene), synthesizes a fatty acid dimer molecule from β-hydroxy fatty acid precursors [56]. The second step, RhlB rhamnosyltransferase chain B (encoded by the *rhlB* gene), produces mono-rhamnolipids by covalently bonding the previously synthesised precursor molecule and dTDP-l-rhamnose [57]. The final step, RhlC rhamnosyltransferase II (encoded by *rhlC* gene), utilises mono-rhamnolipids synthesised by RhlA and RhlB as a substrate, adding a second dTDP-l-rhamnose moiety to produce di-rhamnolipids [58]. The rhamnolipid congener profile produced by *Marinobacter* sp. MCTG107b is predominantly skewed toward the synthesis of di-rhamnolipids, whereas mono-rhamnolipids were only found in much smaller concentrations. Although a prevalence toward di-rhamnolipid synthesis has been observed in *P. aeruginosa*, and to an even greater extent in *Burkholderia* species, the abundance of di-rhamnolipid versus mono-rhamnolipid in *Marinobacter* sp. MCTG107b was significantly higher than that previously observed with other rhamnolipid-producing organisms. In *P. aeruginosa* a single copy of *rhlA* and *rhlB* are located in an operon alongside genes encoding an AHL-mediate quorum sensing system. The *rhlC* gene is located approx. 1 Mbp down stream of this operon [51]. A contrasting arrangement was observed in *B. thailandensis* which has two identical and functional operons containing orthologues of each rhamnolipid synthesis gene (*rhlA*, *rhlB* and *rhlC*), possessing only 40% sequence similarity to those of *P. aeruginosa* [23, 59]. Therefore, *Burkholderia* species can simultaneously express *rhlB* and *rhlC* favouring the immediate addition of the second rhamnosyl group to the produced mono-rhamnolipid [59].

We therefore postulate that a biosynthetic pathway similar to that observed in *Burkholderia* might be present here and accounting for the higher ratio of di-rhamnolipid to mono-rhamnolipid congeners. The biosynthetic pathway of rhamnolipid synthesis by *Marinobacter* sp. has however presented a paradox. Although not shown here, we have carried out additional PCR screening for *rhlA*, *rhlB* and *rhlC* using degenerate primers designed from multiple sequence alignments of both *P. aeruginosa* and *B. thailandensis* sequences. To date, this approach has failed to amplify any DNA sequence which could be involved in rhamnolipid synthesis gene orthologues. Our finding that other similar *Marinobacter* strains fail to synthesise rhamnolipids suggests that the acquisition of rhamnolipid biosynthesis genes by *Marinobacter* sp. MCTG107b may have occurred through lateral gene transfer from an unrelated rhamnolipid producing species, as has been observed previously in other rhamnolipid synthesising bacteria [60]. However, based on the un-relatedness of *Marinobacter* sp. MCTG107b to previously reported rhamnolipid producers and the significant sequence differences between the *P. aeruginosa* and *B. thailandensis* synthases, we conclude that rhamnolipid synthesis in this strain is being catalysed by enzymes with significantly different peptide sequences to either of these other species. This sequence difference is also present at the genetic level, accounting for the reasons why our screening protocol failed [23, 59]. Additionally, the observation of mono-rhamnolipid congeners only possessing a single fatty acid side chain being synthesised by this strain corroborates this conclusion since RhlA in both *P. aeruginosa* and *Burkholderia* species utilises fatty acid dimers as a substrate for rhamnolipid synthesis [56, 59]. To further investigate the mechanisms of rhamnolipid biosynthesis, we recently obtained the complete genome sequence of *Marinobacter* sp. MCTG107b. We are therefore, currently in the process of carrying out comparative genomic analysis with various other *Marinobacter* strains, with the aim of identifying putative genetic candidates for rhamnolipid biosynthesis.

## Conclusions

*Marinobacter* sp. MCTG107b, isolated from the marine environment, has the ability to synthesise a wide variety of rhamnolipid congeners. To the best of our knowledge, rhamnolipid production has not been previously observed in any member of the genus *Marinobacter*. Therefore, the results presented here expand the list of known rhamnolipid producing bacterial taxa to include *Marinobacter*; a genus of marine bacteria that shows little to no association with human pathogenicity. Although the major hurdle of low production yield remains, synthesis of rhamnolipids from novel, non-pathogenic marine species, such as *Marinobacter* sp. MCTG107b, is promising for the scale-up in bioprocessing industry or to provide genetic resources for metabolic engineering for the production of specific rhamnolipid congeners.

## Supplementary information

**Additional file 1: Fig. S1.** Effect of various sea salt concentrations, pH, temperature and nitrogen source on the growth of *Marinobacter* sp. MCTG107b over a period of 96 h. Growth curves show the OD_600_ of strain MCTG107b during growth at different (A) salinities (5.0 to 40 g L^−1^), (B) pH values (4.0 to 8.5), or (C) temperatures (25 °C, 28 °C, 30 °C, 37 °C). **Fig. S2.** HPLC–MS chromatogram for rhamnolipids produced by *Marinobacter* sp. MCTG107b. The MS was operated in the negative mode. Main intensities in the chromatogram were Rha-Rha-C_10_-C_10_ and Rha-Rha-C_10_-C_10_-CH_3_. **Table S1.** Full 2^2^ factorial design with pH and salt as independent variables using surface tension and biosurfactant yield as response variables. Surface tension and biosurfactant yield according to full factorial design after 96 h of shake-flask study of *Marinobacter* sp. MCTG107b. **Table S2.** Analysis of variance (ANOVA) for response variables surface tension and biosurfactant yield by *Marinobacter* sp. MCTG107b.

## Data Availability

All data generated or analysed during this study are included in this published article and its additional files.

## Abbreviations

PAHs: polycyclic aromatic hydrocarbons
MEOR: microbial enhanced oil recovery
*β*-OH-FA: *β*-hydroxy fatty acids
DoE: design of experiments
FFD: full factorial design
gDNA: genomic DNA
EI: emulsification index
SPE: solid phase extraction
HPLC–MS: high performance liquid chromatography–mass spectrometry
NMR: Nuclear Magnetic Resonance
