# Supplementary material for: Biosynthesis of rhamnolipid by a Marinobacter species expands the paradigm of biosurfactant synthesis to a new genus of the marine microflora
Source: Microb Cell Fact. 2019 Oct 10;18:164. doi: 10.1186/s12934-019-1216-8 (PMC6785906; doi:10.1186/s12934-019-1216-8)
Supplement: Supplementary file 1 — Additional file 1: Fig. S1. Effect of various sea salt concentrations, pH, temperature and nitrogen source on the growth of Marinobacter sp. MCTG107b over a period of 96 h. Growth curves show the OD600 of strain MCTG107b during growth at different (A) salinities (5.0 to 40 g L−1), (B) pH values (4.0 to 8.5), or (C) temperatures (25 °C, 28 °C, 30 °C, 37 °C). Fig. S2. HPLC–MS chromatogram for rhamnolipids produced by Marinobacter sp. MCTG107b. The MS was operated in the negative mode. Main intensities in the chromatogram were Rha-Rha-C10-C10 and Rha-Rha-C10-C10-CH3. Table S1. Full 22 factorial design with pH and salt as independent variables using surface tension and biosurfactant yield as response variables. Surface tension and biosurfactant yield according to full factorial design after 96 h of shake-flask study of Marinobacter sp. MCTG107b. Table S2. Analysis of variance (ANOVA) for response variables surface tension and biosurfactant yield by Marinobacter sp. MCTG107b. [file 12934_2019_1216_MOESM1_ESM.docx]

**Additional File 1:**

**
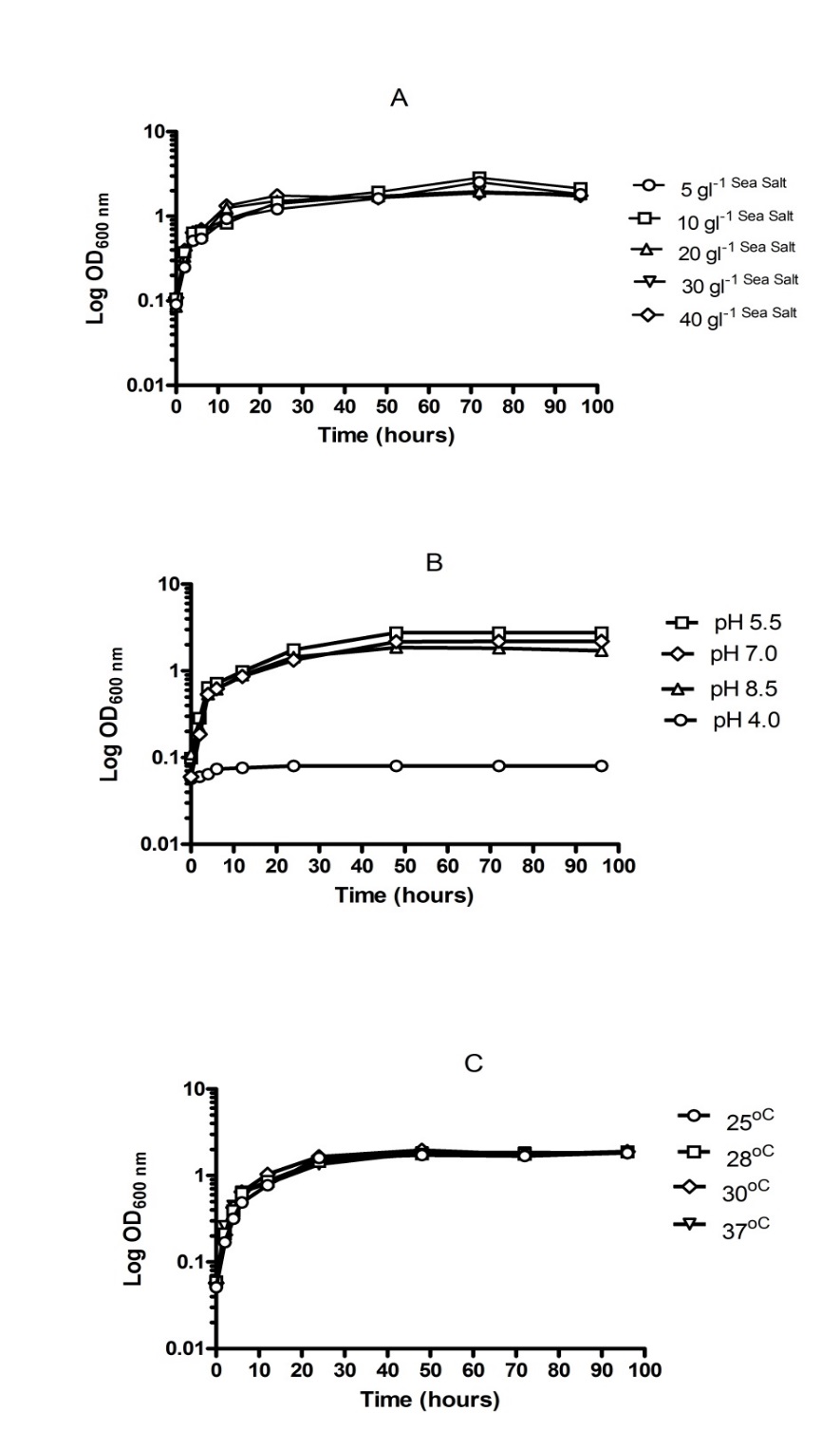
**

**Fig. S1.** Effect of various sea salt concentrations, pH, temperature and nitrogen source on the growth of *Marinobacter* sp. MCTG107b over a period of 96h. Growth curves show the OD_600_ of strain MCTG107b during growth at different (A) salinities (5.0 to 40 g/L), (B) pH values (4.0 to 8.5), or (C) temperatures (25^o^C, 28^o^C, 30^o^C, 37^o^C).


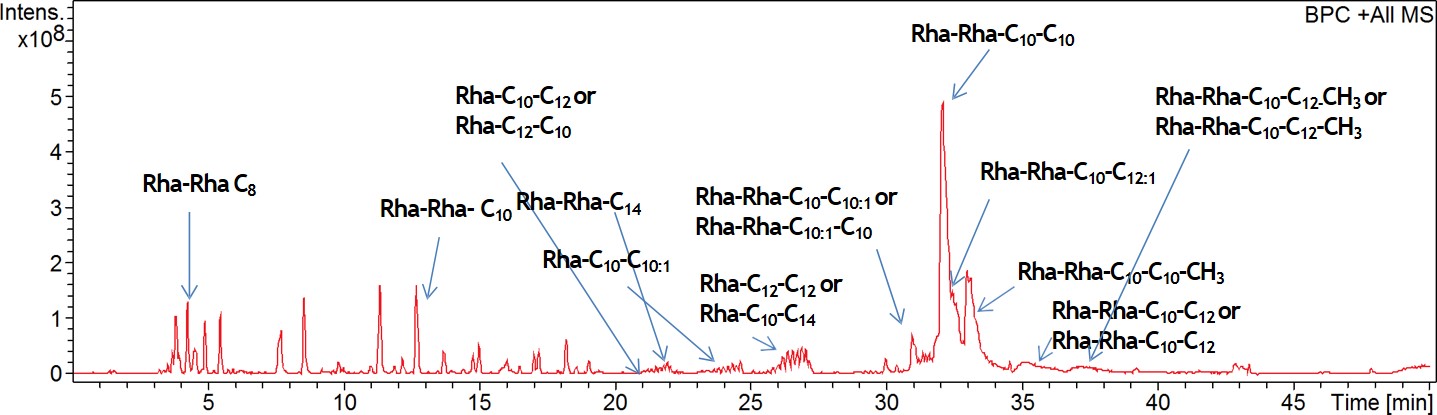


**Fig. S2.** HPLC-MS chromatogram for rhamnolipids produced by *Marinobacter* sp. MCTG107b. The MS was operated in the negative mode. Main intensities in the chromatogram were Rha-Rha-C_10_-C_10_ and Rha-Rha-C_10_-C_10_-CH_3_.

**TABLE S1.** Full 2^2^ factorial design with pH and salt as independent variables using surface tension and biosurfactant yield as response variables. Surface tension and biosurfactant yield were deermined according to full factorial design after 96 h of shake-flask study of *Marinobacter* sp. MCTG107b.

| **Variables** | | **Level** | | | | | | |
| --- | --- | --- | --- | --- | --- | --- | --- | --- |
|  |  | **Low (-1)** | | **Central (0)** | | | **High (+1)** | |
| Sea Salt (gL^-1^) | | 5 | | 22.5 | | | 40 | |
| pH | | 5.5 | | 7.0 | | | 8.5 | |
| **Standard**  **Run** | **Replicate** | | **Central point** | **pH** | **Salts**  **(gL^-1^)** | **ST**  **(mNm^-1^)** | | **BS Yield (mgL^-1^)** |
| 1 | 1 | | 1 | 5.5 | 5.0 | 39 | | 300 |
| 2 | 1 | | 1 | 8.5 | 5.0 | 34.50 | | 340 |
| 3 | 1 | | 1 | 5.5 | 40.0 | 30.80 | | 690 |
| 4 | 1 | | 1 | 8.5 | 40.0 | 32.20 | | 250 |
| 5 | 1 | | 0 | 7.0 | 22.5 | 34.00 | | 400 |
| 6 | 1 | | 0 | 7.0 | 22.5 | 33.80 | | 410 |
| 7 | 1 | | 0 | 7.0 | 22.5 | 33.00 | | 380 |
| 8 | 2 | | 1 | 5.5 | 5.0 | 40.20 | | 290 |
| 9 | 2 | | 1 | 8.5 | 5.0 | 36.10 | | 350 |
| 10 | 2 | | 1 | 5.5 | 40.0 | 30.50 | | 675 |
| 11 | 2 | | 1 | 8.5 | 40.0 | 32.00 | | 240 |
| 12 | 2 | | 0 | 7.0 | 22.5 | 34.00 | | 400 |
| 13 | 2 | | 0 | 7.0 | 22.5 | 33.90 | | 380 |
| 14 | 2 | | 0 | 7.0 | 22.5 | 34.10 | | 400 |

**TABLE S2.** Analysis of variance (ANOVA) for response variables surface tension and biosurfactant yield by *Marinobacter* sp. MCTG107b

| Surface tension^a^ | | | | | |
| --- | --- | --- | --- | --- | --- |
| Factor | Sum of squares | Degrees of freedom | Mean square | F-ratio | P-value |
| pH(1) | 4.06125 | 1 | 4.06125 | 10.3566 | 0.014691 |
| Salts (2) | 73.81125 | 1 | 73.81125 | 188.2254 | 0.00003 |
| 1 by 2 | 16.53125 | 1 | 16.53125 | 42.1562 | 0.000336 |
| Lack of Fit | 1.35375 | 1 | 1.35375 | 3.4522 | 0.105520 |
| Pure Error | 2.74500 | 7 | 0.39214 |  |  |
| Total SS | 98.50250 | 11 |  |  |  |
| Biosurfactant yield^b^ | | | | | |
| Factor | Sum of squares | Degrees of freedom | Mean square | F-ratio | P-value |
| pH(1) | 75078.1 | 1 | 75078.1 | 712.606 | 0.000000 |
| Salts (2) | 41328.1 | 1 | 41328.1 | 392.267 | 0.000000 |
| 1 by 2 | 118828.1 | 1 | 118828.1 | 1127.860 | 0.000000 |
| Lack of Fit | 84.4 | 1 | 84.4 | 0.801 | 0.400563 |
| Pure Error | 737.5 | 7 | 105.4 |  |  |
| Total SS | 236056.3 | 11 |  |  |  |

^a^*R^2^ = 0.95839; Adj. R^2^ = 0.94279; p≤ 0.05-significant at 5% level*

^b^*R^2^ = 0.99652; Adj. R^2^ = 0.99521; p≤ 0.05-significant at 5% level*
